# Preoperative coronal imbalance in degenerative scoliosis: a study on coronal and sagittal spinal-pelvic parameters——a retrospective study

**DOI:** 10.1186/s12891-025-09197-4

**Published:** 2025-10-07

**Authors:** Mei-Fang Wu, Yu-Sheng Bao, Hao Zhang, Yu-Zhi Ning, Zan Chen, Li-Peng Zheng, Fei Lei, Da-Xiong Feng

**Affiliations:** 1 Dazhou Dachuan District People’s Hospital, (Dazhou Third People’s Hospital), No.700, Hanxing North Street, Sanliping Subdistrict, Da Chuan District, Da Zhou, Sichuan Province 635000 PR China; 2 Department of Spinal Surgery, The Affiliated Hospital of Southwest Medical University, No 25 Tai Ping Street, Jiang Yang District, Luzhou, Sichuan Province 646000 PR China

**Keywords:** Coronal imbalance, Pelvic parameters, Sagittal parameters, Degenerative scoliosis

## Abstract

**Objective:**

To investigate the relationship between spinal-pelvic parameters in the coronal and sagittal planes and preoperative coronal imbalance (CIB) in degenerative scoliosis, aiming to prevent preoperative CIB and restore coronal balance(CB) for improved surgical outcomes.

**Methods:**

From May 2018 to May 2024, adult patients who underwent full-length spine imaging, were analyzed at the Southwest Medical University Affiliated Hospital. The inclusion criteria were: (1) availability of clear full-length spinal images in the coronal and sagittal planes that allowed for measurement of relevant parameters; (2) complete demographic information; (3) a major curve angle greater than 10°; and (4) skeletal maturity. Exclusion criteria were as follows : (1) history of previous spinal surgery; (2) pre-existing spinal or pelvic deformities; (3) history of trauma to the spine or pelvis; and (4) history of spinal infectious disease.A total of 162 cases were collected based on the inclusion and exclusion criteria.The general and imaging data of 162 patients were collected. These included the major curve (MC), fractional curve (FC), L5 tilt angle (L5TA), coronal pelvic inclination (CPI), apical vertebra translation (AVT), the number of vertebrae in the primary curve, apical vertebral rotation (AVR), sacral slope (SS), lumbar lordosis (LL), pelvic incidence (PI), pelvic tilt (PT), and sagittal vertical axis (SVA). Pearson correlation analysis and linear regression were employed to assess the relationship of each parameter with preoperative coronal balance distance (CBD). CBD was then converted to a binary variable (Patients with a CBD less than 3.0 cm were categorized into the CB group, while those with a CBD of 3.0 cm or greater were placed in the CIB group.). Univariate screening, multivariate logistic regression, and receiver operating characteristic (ROC) curve analysis were conducted to identify associations between preoperative CIB and the specified parameters.A total of 162 patients were classified into three groups based on the classification criteria of the Gulou Hospital: Type A (120 cases), Type B (25 cases), and Type C (17 cases). The differences in imaging data among the three groups were compared.

**Results:**

Pearson analysis demonstrated that L5TA, CPI, number of vertebrae in the primary curve, LL, SS, and SVA were correlated with preoperative CBD (*p* < 0.05). Moreover, further linear regression indicated that merely L5TA (R² = 0.204, *p* < 0.05), CPI (R² = 0.128, *p* < 0.05), and SVA (R² = 0.172, *p* < 0.05) were substantially associated with preoperative CBD, despite the fact that the relationship was not strictly linear. Multivariate logistic regression and ROC curve analysis revealed that age < 60.5 years was a protective factor against preoperative CIB, while preoperative L5TA > 5.75°, CPI > 3.55°, and SVA > 5.305 cm were risk factors for preoperative CIB. Among the 162 patients, 120 were classified as Type A, 25 as Type B, and 17 as Type C. Significant differences in age and L5TA were observed between the A and C groups. CBD, CPI, and SVA exhibited statistically significant differences between the A group and both the B and C groups, whereas no significant difference was found between the B and C groups.

**Conclusion:**

Preoperative L5TA is an independent risk factor for preoperative CIB. When the preoperative C7PL is located on the convex side of the major curve, L5 tilt becomes more pronounced. In the surgical treatment of DS, leveling the L5 vertebra can help reduce the incidence of postoperative CIB. Patients with degenerative scoliosis (DS) under 60.5 years of age might reduce CIB incidence through enhanced paraspinal muscle strength. Additionally, imbalances in CPI and SVA may contribute to preoperative CIB, and pelvic and sagittal alignment maintenance may offer spinal support essential for preserving coronal balance.

## Introduction

Degenerative scoliosis (DS) is an adult spinal deformity distinguished by a curvature that emerges post-skeletal maturity, independent of other spinal disorders including infections, tumors, congenital anomalies, trauma, or genetic influences. A widely accepted mechanism for DS indicates asymmetric degeneration of the intervertebral discs and facet joints, resulting in uneven disc height reduction, vertebral slippage, and rotation [1, 2]. DS presents as a three-dimensional deformity, affecting the coronal, sagittal, as well as axial planes, which substantially complicates treatment.

Conservative treatment is generally considered the first-line approach for DS, although previous studies have indicated that surgical intervention frequently results in more favorable outcomes [3]. Numerous studies link sagittal imbalance with a decline in health-related quality of life (HRQoL), focusing primarily on sagittal parameters and their relationship with DS. Nonetheless, fewer studies have explored the association between coronal parameters and DS. Notably, research implies that coronal imbalance (CIB) is closely linked to decreased HRQoL [4], as CIB can result in trunk tilt, gait abnormalities, low back pain, and concave-side radiculopathy. Additionally, CIB following surgery is closely linked to a greater risk of instrumentation failure, resulting in a higher revision rate and reduced patient satisfaction.

Consequently, recent research has focused on identifying factors associated with CIB. For instance, Lewis [5] determined that coronal tilt of L4 and L5 is linked to postoperative CIB, and Niu [6] identified an L5 tilt angle ≥ 15° as an independent risk factor for CIB at final follow-up. Furthermore, Shu [7] discovered that a convex C7 plumb line, an upper instrumented vertebra above T6, and rotational displacement of the lower instrumented vertebra were all associated with postoperative CIB risk. Nonetheless, these studies primarily examine postoperative CIB, factors linked to preoperative CIB remain insufficiently explored. Some researchimplies that preoperative CIB may predispose patients to postoperative CIB [8], and ZhangZ [9] indicated that preoperative coronal spinal features could guide surgical correction of CIB. Additionally, studies by Cho [10] and Glassman SD [11] also demonstrate that preoperative CIB is associated with increased rates of instrumentation failure, highlighting it as a risk factor for postoperative complications. These findings underscore the importance of assessing CIB preoperatively to reduce postoperative complications, optimize surgical outcomes, and enhance patients’ quality of life.

Currently, limited research has examined preoperative coronal balance distance (CBD) and factors related to preoperative CIB. Besides, previous studies imply that CIB may be associated with sagittal imbalance and pelvic parameter changes [12, 13], yet the specific relationship remains unclear. Furthermore, studies have indicated that coronal pelvic inclination may contribute to an increase in scoliosis, although the relationship between coronal plane pelvic tilt and CBD remains unclear [14]. Therefore, building on prior research, this study extends the analysis of potential factors by exploring the relationships between preoperative CBD, preoperative CIB, and coronal and sagittal spinal-pelvic parameters. The aim is to support the surgical correction of CIB and the overall maintenance of spinal balance.

## Methods

Data were collected from adult patients who underwent full-length spinal imaging (including bending views) at the Affiliated Hospital of Southwest Medical University between May 2018 and May 2024. The inclusion criteria were: (1) availability of clear full-length spinal images in the coronal and sagittal planes that allowed for measurement of relevant parameters; (2) complete demographic information; (3) a major curve angle greater than 10°; and (4) skeletal maturity. Exclusion criteria were as follows: (1) history of previous spinal surgery; (2) pre-existing spinal or pelvic deformities; (3) history of trauma to the spine or pelvis; and (4) history of spinal infectious disease.

Based on these criteria, a total of 162 cases were included in this study. CIB was diagnosed based on a CBD ≥ 3 cm [15], and the patients were subsequently grouped accordingly. Of these, 42 cases had a CBD ≥ 3.0 cm (CIB group), while 120 cases had a CBD < 3.0 cm (CB group). Based on the coronal plane balance classification for degenerative scoliosis from Gulou Hospital [16], the 162 patients were grouped as follows:

- Type A: C7PL-CSVL within 3.0 cm;
- Type B: C7PL-CSVL ≥ 3.0 cm with C7PL on the concave side of the major curve;
- Type C: C7PL-CSVL ≥ 3.0 cm with C7PL on the convex side of the major curve.

### Radiographic parameters

**Fig. 1:**
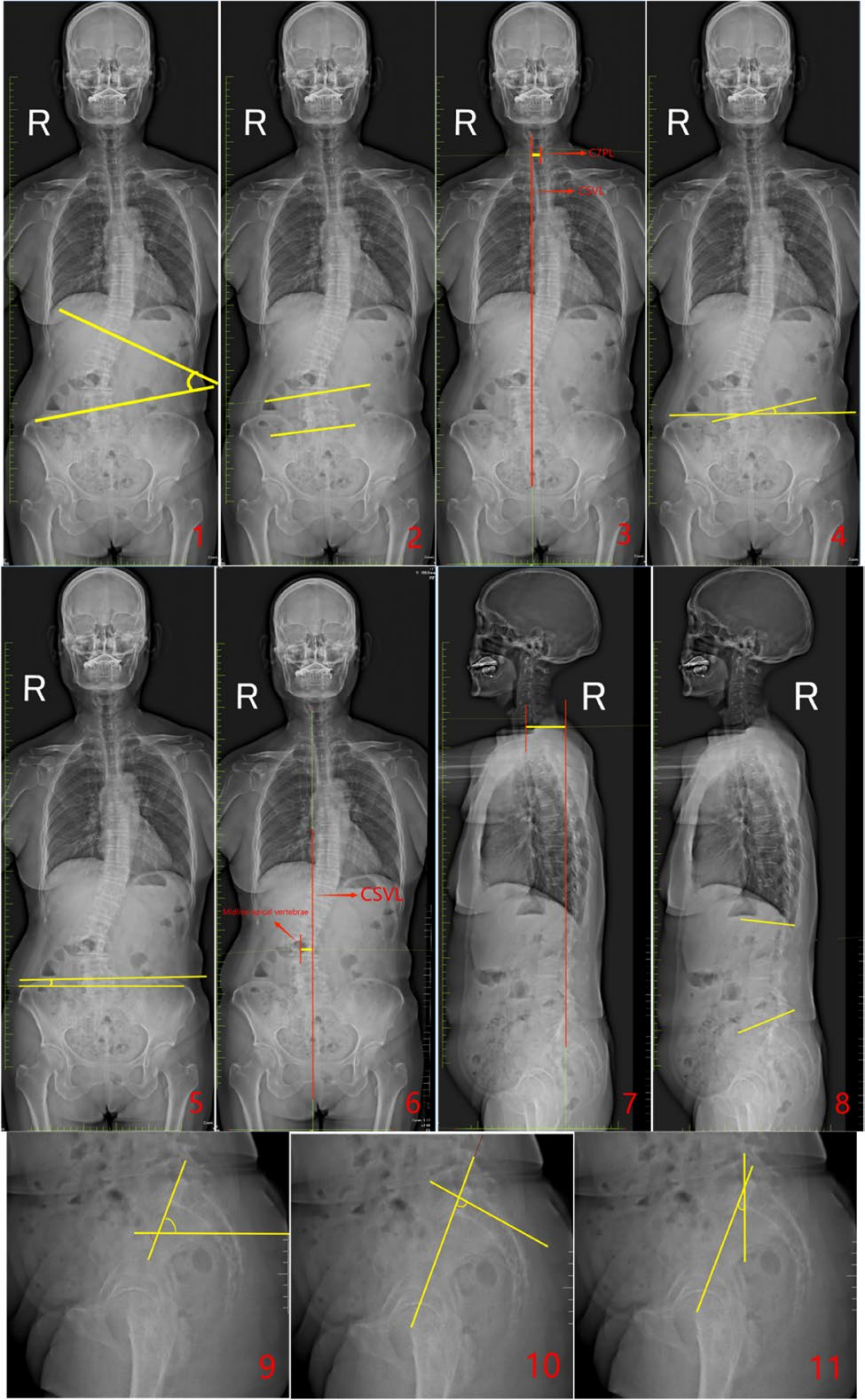
Spine X-ray full length photogrammetry diagram. Figure1-1. The angle between the two yellow lines is the Major Curve (MC). Figure 1−2. The angle between the two yellow lines is the Fractional Curve (FC). Figure 1−3. The yellow line represents the Coronal Balance Distance (CBD). Figure 1−4. The angle between the two yellow lines is the L5 Tilt Angle (L5TA). Figure 1−5. The angle between the two yellow lines is the Coronal Pelvic Inclination (CPI). Figure 1−6. The yellow line indicates the Apical Vertebra Translation (AVT). Figure 1−7. The yellow line represents the Sagittal Vertical Axis (SVA). Figure 1−8. The angle between the yellow lines shows the Lumbar Lordosis (LL). Figure 1−9. The angle between the yellow lines represents the Pelvic Tilt(PT). Figure 1−10. The angle between the two yellow lines demonstrates the Pelvic Incidence (PI). Figure 1−11. The angle between the two yellow lines illustrates the Sacral Slope(SS)

In this study, we utilized X-ray imaging equipment (an ED150L X-ray machine provided by Tshima, Japan) for a full-length spinal examination. All patients underwent standing full-length spinal imaging with their arms supported on a frame, shoulders flexed forward at 30°, and elbows slightly bent (see Fig. 1).

**Fig. 2:**
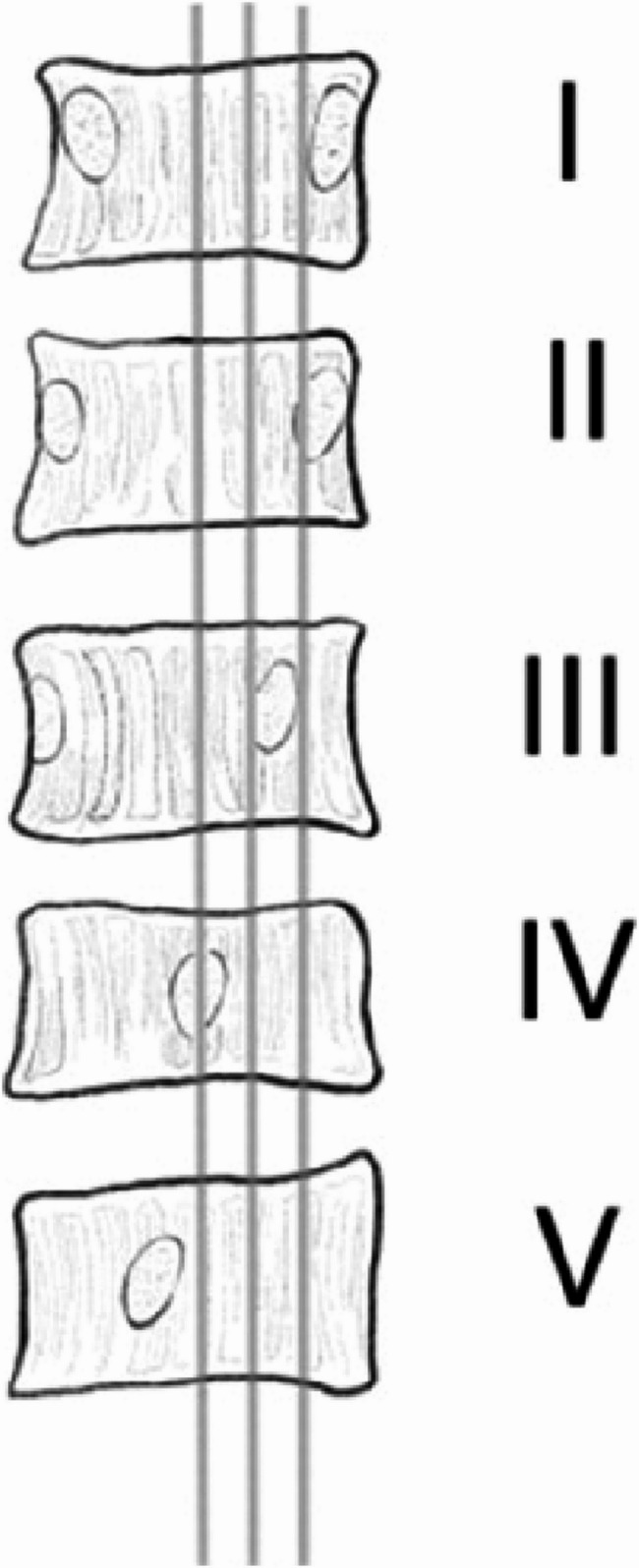
Nash-Moe Method for Measuring Vertebral Rotation Degrees. Degree I: The pedicles are symmetrical. Degree II: The pedicle on the convex side moves toward the midline without surpassing the first grading, while the pedicle on the concave side decreases in size. Degree III: The pedicle on the convex side has shifted to the second grading, while the pedicle on the concave side has disappeared. Degree IV: The pedicle on the convex side has shifted towards the center, while the pedicle on the concave side has vanished. Degree V: The convex side pedicle crosses the midline and approaches the concave side

1. Major Curve (MC): The angle between the tangents of the superior endplate of the uppermost vertebra and the inferior endplate of the lowermost vertebra in the major coronal curve of the spine.
2. Fractional Curve (FC): The angle formed between the tangent of the superior endplate of L4 and the superior endplate of S1 in the lower curve of the spine on the coronal plane.
3. Coronal Balance Distance (CBD): The horizontal distance between the C7 plumb line (C7PL) and the central sacral vertical line (CSVL).
4. Number of main curved vertebrae: The total number of vertebrae involved in the major curve, including the end vertebrae.
5. L5 Tilt Angle (L5TA): The angle formed between the superior endplate of L5 and the line connecting the highest points of both iliac crests (all L5TA values in this study refer to preoperative measurements).
6. Coronal Pelvic Inclination (CPI): The angle between the line connecting the highest points of the iliac crests and the horizontal plane.
7. Apical Vertebral Translation (AVT): The horizontal distance from the midpoint of the apical vertebra (or disc) to the CSVL in the coronal plane.
8. Apical Vertebral Rotation(AVR): Measured using the Nash-Moe method (refer to Fig. 2), classified into grades I, II, III, IV, and V.
9. Pelvic Tilt (PT): In the sagittal plane, the angle between the line connecting the center of the femoral head and the midpoint of the superior endplate of S1, and the vertical line.
10. Pelvic Incidence (PI): The angle formed between the line connecting the center of the femoral head to the midpoint of the S1 superior endplate and the line perpendicular to the superior endplate of S1 in the sagittal plane.
11. Sacral Slope (SS): The angle between the horizontal line and the tangent to the S1 superior endplate in the sagittal plane.
12. Lumbar Lordosis (LL): The angle formed between the superior endplate of L1 and the inferior endplate of L5 in the sagittal plane.
13. Sagittal Vertical Axis (SVA): The horizontal distance between the C7PL and the posterior superior corner of the sacrum in the sagittal plane.

### Statistical analysis

For the relationship between CBD and other continuous variables, linear correlation analysis was performed, and the Pearson correlation coefficient (r) was calculated. The point-biserial correlation coefficient was calculated to assess the relationship between CBD and categorical variables, while Spearman correlation analysis was used to examine the relationship between CBD and ordinal categorical variables. Finally, stepwise multiple linear regression analysis was conducted to identify statistically significant variables (*p* < 0.05) as well as to calculate the coefficient of determination (R²).

For continuous independent variables that followed a normal distribution, a t-test was employed for univariate analysis; for unordered categorical variables, the chi-square test was employed; for ordered categorical variables and continuous variables that did not follow a normal distribution, non-parametric tests were adopted for univariate analysis. Multivariate binary logistic regression analysis was conducted for independent variables that demonstrated statistical significance, with the critical value determined through receiver operating characteristic (ROC) curves. For comparisons among the three groups, data following a normal distribution were analyzed using one-way analysis of variance (ANOVA) with post-hoc tests, while data not following a normal distribution were analyzed using the Kruskal-Wallis test.All analyses were conducted employing SPSS statistical software (version 26.0; IBM). Continuous variables that followed a normal distribution were expressed as mean ± standard deviation (X ± S), and continuous variables that did not follow a normal distribution were presented as median and interquartile range.

## Results

A total of 162 patients were included in this study, comprising 58 males and 104 females.The average age was 66 years (range: 59–73), and the mean BMI was 23.83 ± 3.21 kg/m². The imaging data are summarized in Table 1.

**Table 1:** Statistical analysis of the patients’ imaging parameters

Imaging parameters	X ± S or Median
CBD(cm)	1.48(0.315–2.645)
MC(°)	21.7(14.44–28.96)
FC(°)	8(2.89–13.12)
Number	5(4–6)
L5TA(°)	6.4(2.81–9.99)
LL(°)	26.5(13.25–39.75)
PI(°)	56.18 ± 13.34
PT(°)	24.20 ± 8.59
SS(°)	32.02 ± 11.10
AVT(cm)	2.0 ± 1.32
CPI(°)	1.8(0.9–2.7)
SVA(cm)	3.81(1.23–6.39)

*Abbreviations:* *CBD* Coronal Balance Distance, *MC* Major Curve, *FC* Fractional Curve, *L5TA* L5 Tilt Angle, *CPI* Coronal Pelvic Inclination, *AVT* Apical Vertebral Translation, *LL* Lumbar Lordosis, *PI* Pelvic Incidence, *PT* Pelvic Tilt, *SS* Sacral Slope, *SVA* Sagittal Vertical Axis, *Number* Number of major curved vertebrae. (The overall median CBD value includes all subgroups, with both Type B and Type C subgroups having CBD values ≥ 3 cm)

The point-biserial correlation coefficient between CBD and gender was 0.128, with a P-value of 0.104 (greater than 0.05), suggesting no significant correlation between the two variables. Spearman correlation analysis revealed that the correlation coefficient (rs) between AVR and CBD was − 0.104, with *p* = 0.188, implying no correlation between these variables.

**Fig. 3:**
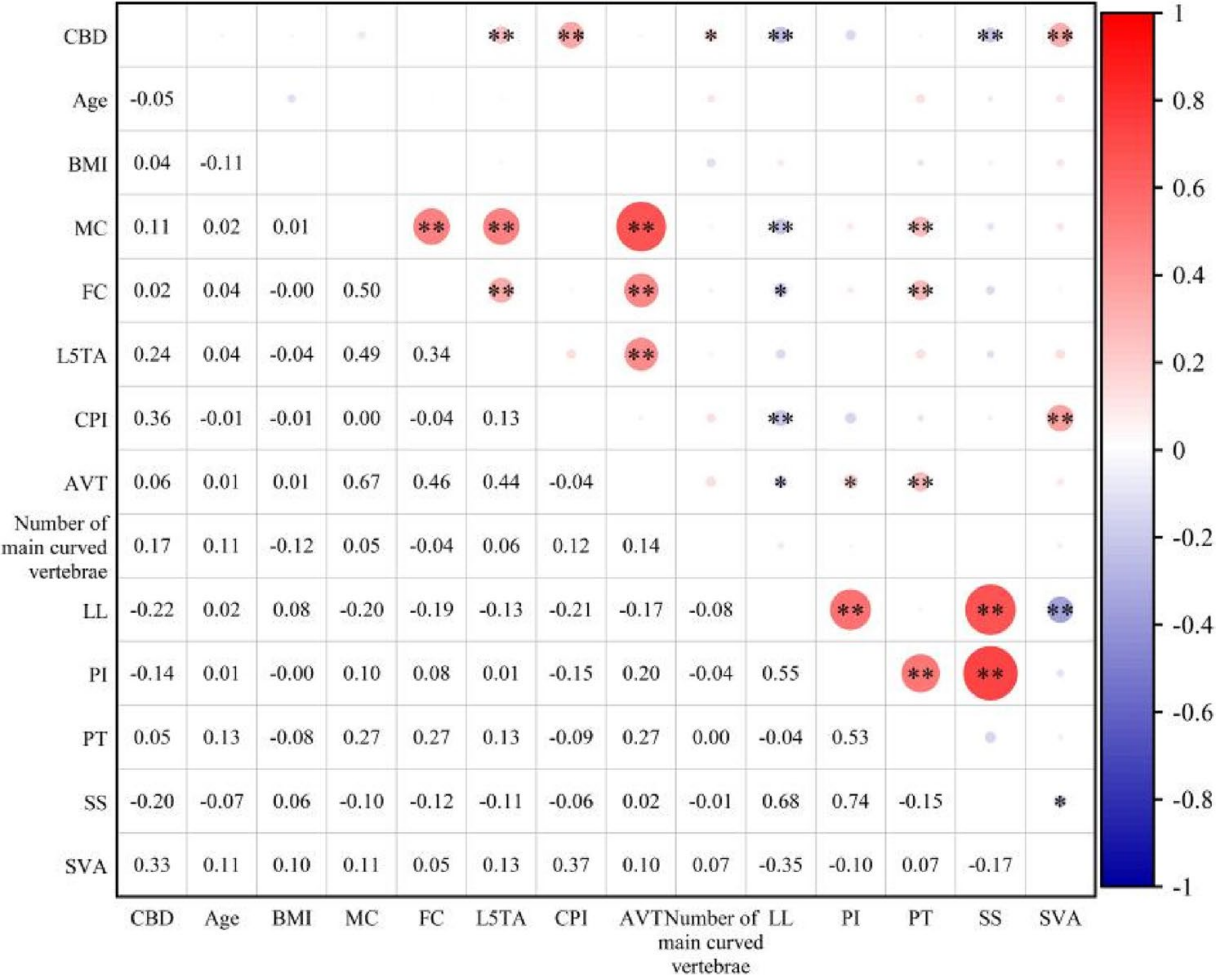
Pearson Correlation Heatmap.** indicates significance at the 0.01 level (two-tailed, *p* < 0.01); * indicates significance at the 0.05 level (two-tailed, *p* < 0.05). Abbreviations: BMI, Body Mass Index; CBD, Coronal Balance Distance; MC, Major Curve; FC, Fractional Curve; L5TA, L5 Tilt Angle; CPI, Coronal Pelvic Inclination; AVT, Apical Vertebral Translation; LL, Lumbar Lordosis; PI, Pelvic Incidence; PT, Pelvic Tilt; SS, Sacral Slope; SVA, Sagittal Vertical Axis

Pearson correlation analysis demonstrated substantial correlations between L5TA, CPI, the number of major curve vertebrae, LL, SS, and SVA with CBD (*p* < 0.05). Significant correlations were also determined among CPI, LL, and SVA. No significant correlations were found between AVT, MC, FC, PI, and CBD (refer to Table 2; Fig. 3).

**Table 2:** Pearson correlation analysis

*r*	BMI (kg/m2)	Age (°)	MC (°)	FC (°)	L5TA (°)	CPI (°)	AVT (cm)	Number	LL (°)	PI (°)	PT (°)	SS (°)	SVA(cm)
CBD(cm)	0.044	−0.051	0.105	0.015	0.241**	0.357**	0.056	0.174*	−0.220**	−0.137	0.054	−0.202**	0.327**

*Abbreviations:* *CBD* Coronal Balance Distance, *BMI* Body Mass Index, *MC* Major Curve, *FC* Fractional Curve, *L5TA* L5 tilt angle, *CPI* Coronal pelvic inclination, *AVT* apical vertebra translation, *LL* Lumbar lordosis, *PI* Pelvic Incidence, *PT* Pelvic Tilt, *SS* Sacral Slope, *SVA* Sagittal Vertical Axis, *Number* Number of main curved vertebrae

** indicates significance at the 0.01 level (two-tailed, *p* < 0.01); * indicates significance at the 0.05 level (two-tailed, *p* < 0.05)

**Fig. 4:**
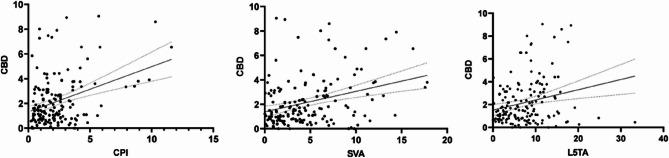
Linear Correlation Scatter Plots of CPI, SVA, L5TA, and CBD. Abbreviations: CBD, Coronal Balance Distance; L5TA, L5 Tilt Angle; CPI, Coronal Pelvic Inclination; SVA, Sagittal Vertical Axis

Further stepwise linear regression analysis indicated that L5TA (R²=0.204, *p* < 0.05), CPI (R²=0.128, *p* < 0.05), and SVA (R²=0.172, *p* < 0.05) substantially influenced CBD (scatter plot demonstrated in Fig. 4).

**Fig. 5:**
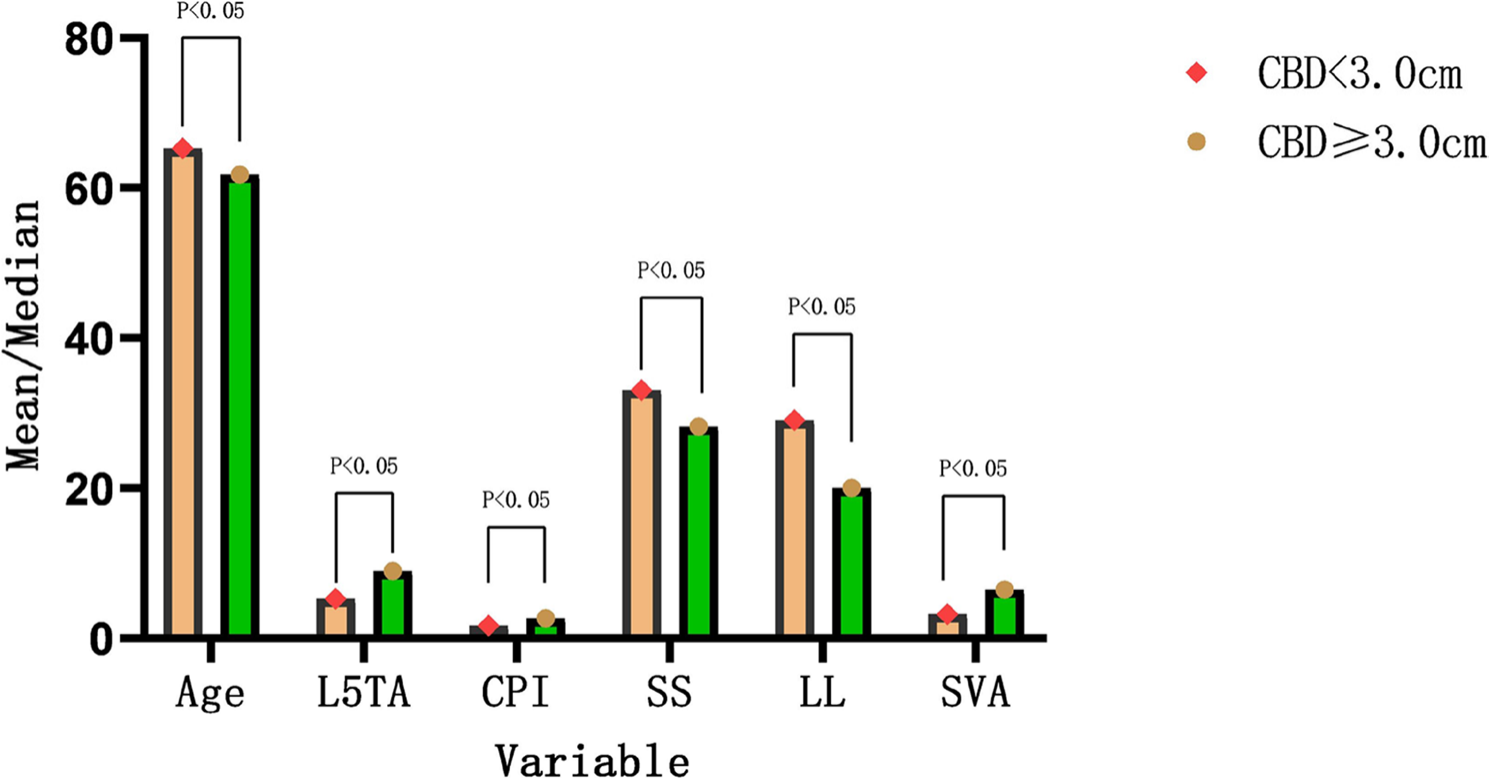
Bar Chart of Univariate Analysis. Abbreviations: L5TA, L5 Tilt Angle; CPI, Coronal Pelvic Inclination; LL, Lumbar Lordosis; SS, Sacral Slope; SVA, Sagittal Vertical Axis

Based on the size of CBD, among the 162 patients, 120 exhibited CB, while 42 had CIB. Univariate analysis demonstrated statistically significant differences in Age, L5TA, CPI, SS, LL, and SVA between the two groups (*p* < 0.05) (refer to Table 3; Fig. 5). There was no statistically significant difference in gender between the two groups (*p* = 0.138), and AVR also illustrated no statistically significant difference between the two groups (refer to Table 4).

**Table 3:** Univariate screening of variables

	CB group (*n* = 120)	CIB group (*n* = 42)	t or z	*p*
Age	65.33 ± 9.81	61.79 ± 9.79	2.018	0.045
*MC(°)*	22.2(16.5 ~ 30.85)	21.3(16.68 ~ 32.35)	−0.05	0.960
FC*(°)*	8(3.525 ~ 14.025)	8.5(3.2 ~ 12.8)	−0.061	0.951
BMI(*kg/m*^*2*^)	23.58 ± 0.30	24.55 ± 0.44	−1.701	0.091
Number	5(4 ~ 6)	5(4.75 ~ 7)	−1.704	0.088
L5TA*(°)*	5.3(2.4 ~ 9.1)	9(6.05 ~ 11.925)	−3.662	0.000
CPI*(°)*	1.7(0.925 ~ 2.6)	2.7(1.45 ~ 4.425)	−3.237	0.001
AVT*(cm)*	1.765(1.0925 ~ 2.4675)	1.97(0.505 ~ 3.5525)	−0.204	0.838
PI(*°*)	57.19 ± 12.82	53.27 ± 14.50	1.65	0.101
PT*(°)*	22.9(17.925 ~ 28.6)	25(17.4 ~ 31.05)	−0.612	0.541
SS*(°)*	33.05(22.925 ~ 41.1)	28.3(22.15 ~ 36.325)	−2.125	0.034
LL*(°)*	29.15(16.4 ~ 41.4)	20.1(6.9 ~ 34.3)	−2.761	0.006
SVA*(cm)*	3.25(1.3925 ~ 5.585)	6.525(2.505 ~ 9.9075)	−3.499	0.000

*Abbreviations:* *BMI* Ballistic Missile Intercept, *MC* Major Curve, *FC* Fractional Curve, *L5TA* L5 Tilt Angle, *CPI* Coronal Pelvic Inclination, *AVT* Apical Vertebral Translation, *LL* Lumbar Lordosis, *PI* Pelvic Incidence, *PT* Pelvic Tilt, *SS* Sacral Slope, *SVA* Sagittal Vertical Axis, *Number* Number of main curved vertebrae

**Table 4:** Chi-Square Test of AVR and CBD

	Apical vertebral rotation					χ^2^	*p*
	I	II	III	IV	V		
CB group (*n* = 120)	53 (44.1%)	41 (34.2%)	20 (16.7%)	5 (4.2%)	1 (0.8%)	0.541	0.589
CIB group (*n* = 42)	20 (47.6%)	15 (35.7%)	5 (11.9%)	1 (2.4%)	1 (2.4%)		

**Fig. 6:**
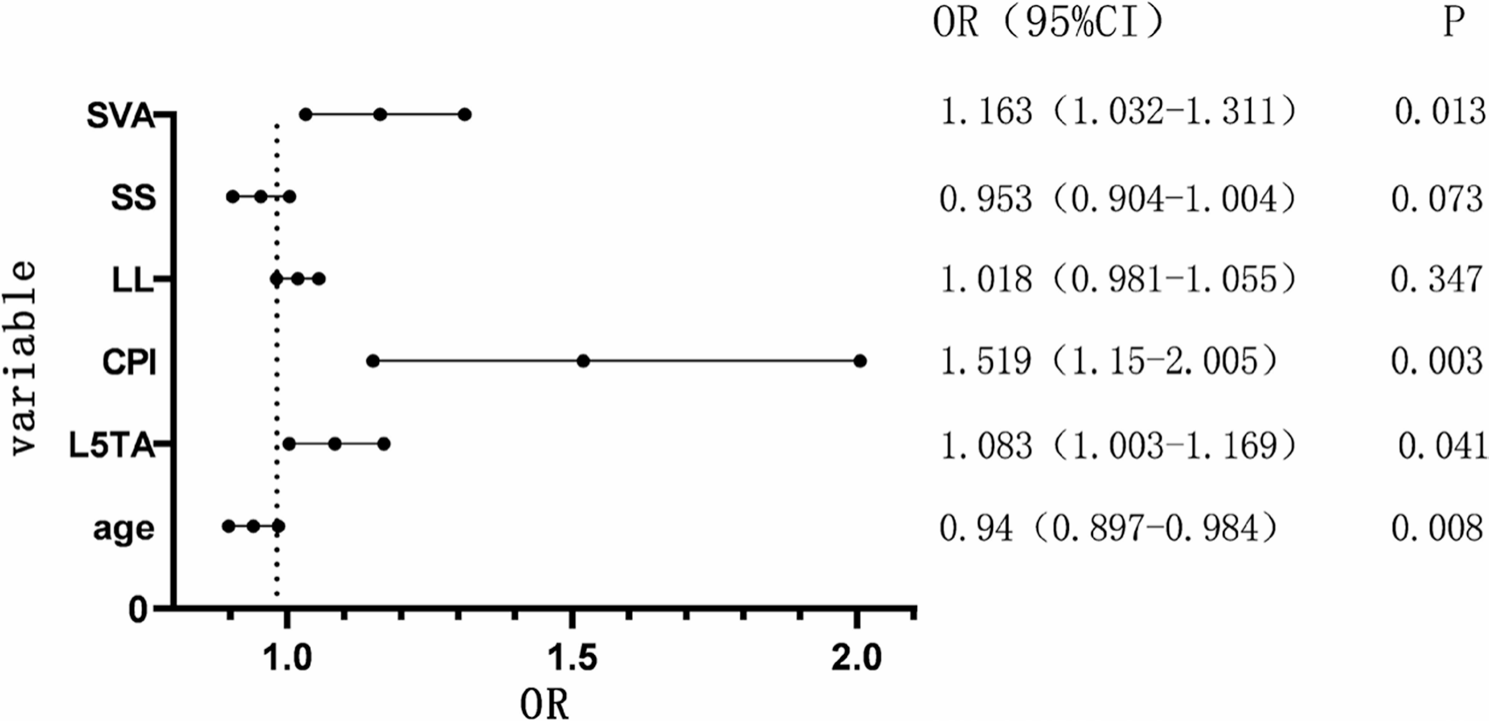
Forest Plot of Binary Logistic Regression Analysis. Abbreviations: L5TA, L5 Tilt Angle; CPI, Coronal Pelvic Inclination; LL, Lumbar Lordosis; SS, Sacral Slope; SVA, Sagittal Vertical Axis

**Fig. 7:**
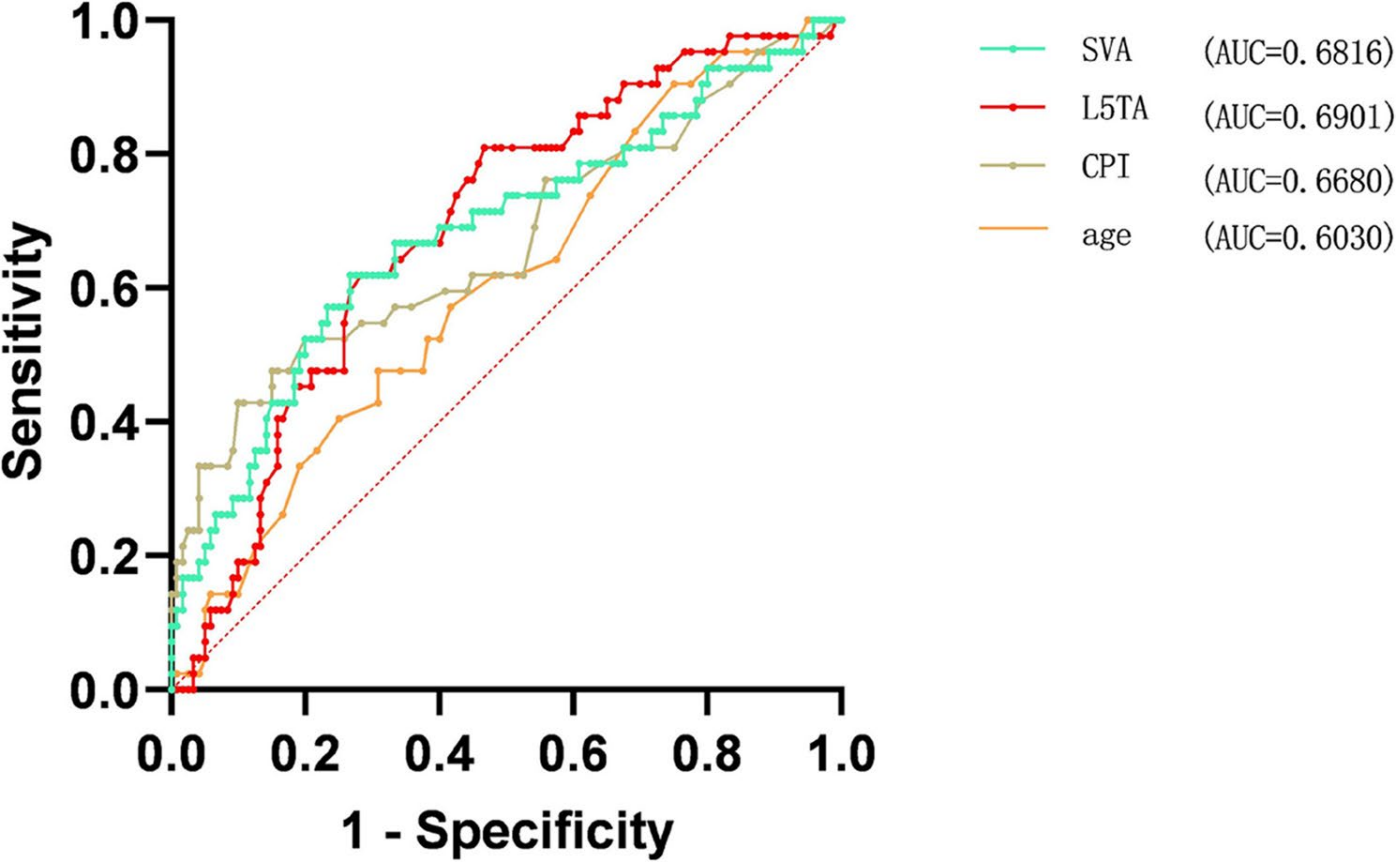
ROC Curve. Abbreviations: L5TA, L5 Tilt Angle; CPI, Coronal Pelvic Inclination; SVA, Sagittal Vertical Axis

Multivariate binary logistic regression analysis was conducted on the statistically significant variables (collinearity diagnostics confirmed all variance inflation factors were < 5), revealing that Age, L5TA, CPI, and SVA are factors influencing preoperative CIB (*p* < 0.05) (refer to Table 5, and forest plot demonstrated in Fig. 6). ROC curve analysis revealed the following AUC (Area Under the Curve) values: age (AUC = 0.40), L5TA (AUC = 0.69), SVA (AUC = 0.68), and CPI (AUC = 0.67). The results shown in Fig. 7 indicate that age is a protective factor for CIB. Patients with DS who are under the age of 60.5 are less likely to experience CIB. Additionally, L5TA > 5.75°, CPI > 3.55°, and SVA > 5.305 cm are risk factors for the occurrence of CIB in patients with DS.

**Table 5:** Multivariate binary logistic regression analysis

	B	Standard error	Wald χ^2^	OR	*p*
Age	−0.062	0.024	7.005	0.94	0.008
L5TA(°)	0.08	0.039	4.172	1.083	0.041
CPI(°)	0.418	0.142	8.673	1.519	0.003
SS(°)	−0.048	0.027	3.224	0.953	0.073
LL(°)	0.017	0.019	0.883	1.018	0.347
SVA(cm)	0.151	0.061	6.114	1.163	0.013

*Abbreviations:* *L5TA* L5 Tilt Angle, *CPI* Coronal Pelvic Inclination, *LL* Lumbar Lordosis, *SS* Sacral Slope, *SVA* Sagittal Vertical Axis

Comparison among the three groups of Type A, B, and C patients is shown in Table 6. Among the 162 patients, 120 were classified as Type A, 25 as Type B, and 17 as Type C.Type A patients were older than Type C patients (*p* = 0.042), while Type C patients had significantly higher L5TA than Type A patients (*p* < 0.001).The CPI, SVA, and CBD values were significantly lower in Type A patients compared to Type B and Type C patients (CBD: A vs. B: *p* < 0.001, A vs. C: *p* < 0.001; CPI: A vs. B: *p* = 0.003, A vs. C: *p* = 0.042; SVA: A vs. B: *p* = 0.006, A vs. C: *p* = 0.031).The SS and LL values in Type A patients were higher than those in Type B patients (SS: *p* = 0.014;LL:*p* = 0.024).There were no statistically significant differences in the imaging parameters between the B and C groups.

**Table 6:** Comparison of imaging parameters among DS patients with preoperative coronal plane alignments of types A, B, and C

	A	B	C	A vs. B (*p*)	A vs. C (*p*)	B vs. C (*p*)
N	120	25	17	-	-	-
Age	67(60.25–73.75)	68(60.25–75.75)	58(53–63)	1	0.042	0.399
BMI(Kg/m^2^)	23.58 ± 3.29	24.36 ± 2.63	24.83 ± 3.23	>0.05	>0.05	>0.05
CBD(cm)	1.07(0.53–1.62)	4.42(2.7–6.14)	3.76(3.11–4.41)	<0.001	<0.001	1
MC(°)	22.2(15.03–29.38)	19.6(12.6–26.6)	23.7(15.5–31.9)	>0.05	>0.05	>0.05
FC(°)	8(2.75–13.25)	6.8(2.8–10.8)	11.4(3.55–19.25)	>0.05	>0.05	>0.05
Number	5(4–6)	6(5–7)	5(3.5–6.5)	>0.05	>0.05	>0.05
L5TA(°)	5.3(1.95–8.65)	8(4.38–11.63)	10.9(8.2–13.6)	0.177	<0.001	0.173
LL(°)	29.15(16.65–41.65)	12.9(2.35–23.45)	22.9(9.45–36.35)	0.024	0.476	1
PI(°)	57.19 ± 12.82	50.25 ± 13.66	57.71 ± 14.95	>0.05	>0.05	>0.05
PT(°)	22.9(17.56–28.24)	24.7(17.4–32)	26(18.13–33.88)	>0.05	>0.05	>0.05
SS(°)	33.15 ± 11.12	27.14 ± 9.79	31.28 ± 11.28	0.014	0.51	0.231
AVT(cm)	1.77(1.08–2.46)	1.70(1.02–2.37)	2.06(1.64–2.47)	>0.05	>0.05	>0.05
CPI(°)	1.7(0.86–2.54)	3.1(1-5.2)	2.7(1.2–4.2)	0.003	0.042	0.685
SVA(cm)	3.25(1.16–5.35)	7.07(2.61–11.53)	6.33(3.32–9.34)	0.006	0.031	1

*Abbreviations:* *BMI* Body Mass Index, *CBD* Coronal Balance Distance, *MC* Major Curve, *FC* Fractional Curve, *L5TA* L5 Tilt Angle, *CPI* Coronal Pelvic Inclination, *AVT* Apical Vertebral Translation, *LL* Lumbar Lordosis, *PI* Pelvic Incidence, *PT* Pelvic Tilt, *SS* Sacral Slope, *SVA* Sagittal Vertical Axis, *Number *Number of main curved vertebrae

## Discussion

Degenerative scoliosis (DS) refers to a spinal deformity resulting from intervertebral disc and facet joint degeneration after skeletal maturity, which may result in symptoms including low back pain, lower limb pain, and gait abnormalities over time. The surgical treatment of DS has long been a focus of spine surgeons, as restoring overall coronal, sagittal, and axial balance is challenging due to the complexity of the three-dimensional spinal structures. The interrelationships among these balances further complicate surgical treatment, which makes detailed preoperative planning particularly crucial. Moreover, research indicates that postoperative coronal imbalance (CIB) may increase the likelihood of surgical revision [17], and some studies suggest that preoperative CIB may contribute to the development of postoperative CIB. Previous research has demonstrated that a preoperative coronal balance distance (CBD) exceeding 4 cm can affect patients’ quality of life and is a risk factor for postoperative internal fixation failure [11].

Consequently, preoperative CIB represents a significant concern. Several relevant studies have offered valuable insights; for instance, Zhang et al. [9] determined that L4 tilt angle is an independent predictor of preoperative CBD, while Schwender’s research [18] indicated a significant correlation between a stiffer lumbar-sacral curve and preoperative CIB in adolescent idiopathic scoliosis. Furthermore, literature has reported a correlation between preoperative CIB and sagittal imbalance [12]. Nanshan Ma [19] illustrated that there was no statistically significant correlation between CIB and the number of degenerated vertebrae. These studies provide valuable insights into the influencing factors of preoperative CBD and its clinical significance. However, further research analyzing the relationship between preoperative CBD, coronal and sagittal spinal-pelvic parameters, along with additional variables, is needed.

Hence, factors potentially related to preoperative CBD were included, and correlation studies were conducted, which indicated a relationship between preoperative CBD and L5 tilt angle (L5TA), coronal pelvic inclination (CPI), lumbar lordosis (LL), sacral slope (SS), sagittal vertical axis (SVA), and the number of main curve vertebrae. Some studies have figured out that in patients with similar curves, those with greater apical vertebral rotation and fewer affected vertebrae are more likely to experience coronal imbalance in the group with certain preoperative factors, as indicated by the analysis [20]. Comparably, our findings demonstrated a correlation between CBD and the number of main curve vertebrae; nonetheless, in contrast, this relationship was negative. It is hypothesized that the accumulation of tilting or slippage across multiple vertebrae may serve as a primary cause for the increased CBD, with the involvement of more vertebrae potentially leading to a larger CBD. Further linear regression analysis revealed that only CPI, SVA, and L5TA were significant influencing factors for CBD (*P* < 0.05). Nonetheless, the low model fit indicates a correlation between CPI, SVA, L5TA, and CBD, which may not represent a simple linear relationship. Thus, CBD was converted into a binary variable for multifactorial regression analysis to further examine the relationships between the factors.

In this study, a CBD of ≥ 3 cm was taken as the critical value for CIB as defined by Gulou Hospital. Besides, the 162 DS patients were divided into two groups and converted into a binary variable, with 25.92% of patients classified as having CIB (CBD ≥ 3 cm). Moreover, multivariate logistic regression analysis indicated that an age of < 60.5 years serves as a protective factor for CIB. Research by Liao et al. [21] determined that after the age of 50, the ratio of the cross-sectional area of the paravertebral muscles to that of the vertebral body gradually correlates more strongly with spinal-pelvic parameters (SS and LL). Additionally, other studies have illustrated that paravertebral muscle degeneration in individuals over 60 is substantially associated with spinal-pelvic parameters [22], which supports the conclusion that paravertebral muscles play a vital role in maintaining overall spinal balance [23]. Notably, the impact of aging on muscle function is generally more pronounced in females than in males, with elderly women typically exhibiting lower skeletal muscle mass compared to men [24]. This disparity may be one reason why DS is more common in women; nonetheless, it was revealed in our study that no significant correlation exists between gender and CBD or CIB. This indicates that, although DS is more prevalent in females, no association is found between gender and coronal balance. The ROC curve results from our study imply that age is a protective factor for CIB, with DS patients younger than 60.5 years less likely to exhibit CIB. It is proposed that the greater muscle strength of the lumbar region in DS patients under 60.5 years, compared to those over this age, might be the reason for this protective factor. Therefore, this highlights the importance of considering age as a factor influencing spinal health in clinical treatment.

Since postoperative CIB outcomes are a primary concern for clinicians, researchers have focused on investigating its risk factors. Numerous studies have identified risk factors for postoperative CIB, and we found that preoperative L5TA and CIB are significant predictors of postoperative CIB [5, 8]. This study indicates that L5TA is a risk factor for preoperative CIB. DS patients with a preoperative L5TA > 5.75° are at a higher risk of developing preoperative CIB, and a statistically significant difference in L5TA was observed between the CB and CIB groups. As a component of the spinal base, the tilt of L5 indicates an inclination in the spinal base, which subsequently contributes to the development of CIB.The leveling of L5 directly affects the sequence of upper fusion and fixation. Patients with an improper selection of the distal fixation segment may develop postoperative CIB, with the most common error being the selection of L3 or L4 as the distal fixation vertebra [25]. Therefore, we recommend selecting L5 as the distal fixation vertebra. Leveling L5 is an effective strategy to reduce the incidence of postoperative CIB, while also preserving lumbar-sacral mobility and maintaining the spine’s capacity for spontaneous compensation.However, if the L5TA exceeds 15°, or in cases of L5/S1 spondylolisthesis, a history of L5 laminectomy, or severe degeneration of the L5/S1 intervertebral disc, pelvic fixation should be considered [26–29]. However, Bao et al. [30] reported that postoperative CIB with pelvic fixation may fail to achieve spontaneous compensation during follow-up, resulting in permanent decompensation. It is important to note that if L5 tilt is not adequately corrected, there remains a risk of postoperative CIB, even with distal fixation extending to S1 or S2 [31]. Furthermore, comparison of imaging parameters among Type A, B, and C DS patients revealed that L5TA was more pronounced in patients with C7PL located on the convex side of the major curve.Additionally, we observed that DS patients with Type C coronal plane alignment had a larger FC, which aligns with the findings of Theologis [32]. Preoperative FC curvature is commonly regarded as a compensatory mechanism for scoliosis [33], while a high preoperative L5TA may result from compensation associated with Type C coronal alignment.L5, as the transitional vertebra between the spine and pelvis, forms the horizontal base of the spine. An increase in L5TA reflects a greater inclination of the spinal base, which may worsen preoperative coronal plane tilt.Therefore, we propose that a vicious cycle exists between preoperative Type C coronal alignment abnormalities and L5 tilt. Additionally, preoperative L5 tilt can also influence postoperative coronal plane alignment.Preoperative L5 tilt indicates rigidity of the lumbosacral compensatory curve [5]. In cases of lumbosacral curve rigidity, correcting only the major curve without addressing this rigidity may hinder spontaneous spinal compensation, potentially exacerbating coronal plane imbalance [33]. This may be one of the reasons why patients with preoperative C7PL positioned on the convex side of scoliosis are at a higher risk of developing postoperative CIB.Therefore, in DS patients with a C-shaped coronal alignment, reducing the L5TA during surgery may help lower the incidence of postoperative CIB. We recommend correcting the FC first during surgery. Particular attention should be given to horizontalizing the L5 to restore the spine’s horizontal alignment, followed by the correction of the major curve.Both Bao [34] and Obeid [35] argue that for patients with C7PL on the convex side of the major curve, it is more crucial to address the lumbosacral segmental curve. During posterior correction, the interbody lumbar fusion technique via the intervertebral foramen can be used to correct the lumbosacral compensatory curve in C-shaped patients. Alternatively, asymmetric three-column osteotomy of L5 or unilateral support techniques may be employed to restore the horizontal alignment of L5, thereby aiding in the restoration of coronal balance. For patients with A-type coronal alignment, correcting only the major curve typically results in a lower risk of postoperative coronal imbalance. However, it is crucial to consider the preoperative L5TA and avoid excessive correction of the major curve. L5 tilt can lead to pelvic obliquity, and over-correcting the major curve may disrupt the natural coronal balance mechanism, potentially leading to the development of CIB. Similar to A-type coronal alignment, for patients with B-type coronal alignment, it is essential to consider the preoperative L5TA and avoid over-correcting the major curve. Failure to do so may exacerbate CIB and potentially result in a shift to C-type coronal alignment.

Several studies have indicated that preoperative CIB is associated with pelvic parameters [13, 36], and the findings of this study are consistent with these, demonstrating that CPI, a coronal pelvic parameter, significantly impacts CIB. It is suggested that CPI reflects the inclination of the pelvis in the coronal plane. The ilium connects to the spine via the sacroiliac joint; if CPI > 0, it indicates that the sacrum may likewise be inclined in the coronal plane. Such inclination may cause tilting of the spinal base, which could potentially trigger CIB. DS patients with CPI > 3.55° are more likely to exhibit or develop CIB. Therefore, changes in CPI not only influence pelvic stability but may also have a significant impact on the overall health of the spine. Our results are consistent with prior studies [12] that determined a notable correlation between preoperative CIB and changes in sagittal balance parameters, with DS patients having SVA > 5.305 cm being more likely to develop or experience CIB. It is asserted that proper sagittal balance offers improved support for the spine, allowing it to maintain symmetry in the coronal plane. Nonetheless, our findings also reveal a correlation between CPI and SVA, indicating an essential interplay between the two in spinal health. Given that DS is a three-dimensional structural deformity, the complex interactions between pelvic parameters, sagittal parameters, and coronal parameters inevitably lead to collinearity. This collinearity can complicate the individual analysis of each parameter’s effects in clinical assessment and research, underscoring the significance of considering multidimensional parameters in comprehensive evaluations of spinal health.

This study provides significant clinical implications: First, preoperative evaluation should particularly focus on high-risk patients with L5TA > 5.75° or CPI > 3.55°, for whom a more systematic coronal balance assessment protocol is recommended. Second, for patients with Type C coronal alignment, special attention should be paid to the L5TA parameter. Notably, in Type C patients presenting with concurrent L5 tilt, surgical planning should prioritize correction strategies for the lumbosacral compensatory curve. The findings of this study primarily apply to the natural history assessment of patients with newly DS. By establishing a parameter correlation system unaffected by surgical intervention, this research provides a theoretical foundation for subsequent interventional studies. Based on the retrospective findings of this study, we plan to conduct a multicenter prospective cohort study stratifying DS patients into two groups (L5TA ≤ 5.75° vs. L5TA >5.75°) with 1–3 years of follow-up to systematically validate the clinical predictive value of this critical threshold by comparing intergroup differences in CBD progression patterns.

### Limitations

This study has several important limitations that warrant careful consideration: First, the sample size for CBD ≥ 3.0 cm was small, and all samples were collected from a single hospital. This single-source sampling may limit the generalizability of the findings. Second, there are interactions between certain variables, and although univariate screening was employed to minimize bias, collinearity effects are still unavoidable. Finally, this study did not investigate the correlation between clinical symptoms and radiographic parameters. While our findings confirm significant associations between spinopelvic parameters (L5TA, CPI) and coronal imbalance, the clinical correlations between these radiographic measurements and patient symptomatology warrant further investigation. Future research should incorporate validated quality-of-life measures (e.g., Visual Analog Scale for pain, Oswestry Disability Index for disability, Scoliosis Research Society-22 Questionnaire for scoliosis-specific outcomes) to establish quantitative relationships between radiographic parameters and clinical manifestations.

## Conclusion

Age < 60.5 years is a protective factor against CIB, and DS patients who do not develop CIB may slow the progression of CBD by strengthening the paravertebral muscles. Besides, L5TA may serve as an independent risk factor closely related to the occurrence of CIB. A vicious cycle may exist between the inclination of L5 and the C-shaped coronal alignment, and their combined effect could potentially increase the incidence of postoperative CIB.L5 inclination may be one of the factors contributing to the increased incidence of postoperative CIB in DS patients with C-shaped coronal alignment.Selecting the L5 vertebra as the distal fixation level and leveling the L5 May help reduce the incidence of postoperative CIB, while preserving some compensatory function of the spinal base. There is a correlation between CPI, SVA, and CIB, implying that an inclination in CPI May trigger CIB. Implementing pelvic corrective exercises to bring the CPI closer to 0 degrees on the condition that an inclination is observed may assist in reducing the incidence of CIB. Additionally, SVA is identified as a risk factor for preoperative CIB, and keeping SVA within the normal range may offer adequate spinal support to maintain coronal balance. Hence, during surgery, restoring coronal balance while also maintaining sagittal balance may assist in minimizing the occurrence of postoperative CIB.

## Data Availability

Data cannot be shared openly but are available on request from authors.

## Abbreviations

AVT: Apical Vertebral Translation
AVR: Apical Vertebral Rotation
AUC: Area Under the Curve
CB: Coronal Balance
CBD: Coronal Balance Distance
CIB: Coronal Imbalance
CPI: Coronal Pelvic Inclination
C7PL: C7 Plumb Line
CSVL: Center Sacral Vertical Line
DS: Degenerative Scoliosis
FC: Fractional Curve
HRQoL: Health-related quality of life
LL: Lumbar Lordosis
L5TA: L5 Tilt Angle
MC: Major Curve
PI: Pelvic Incidence
PT: Pelvic Tilt
ROC Curve: Receiver Operating Characteristic Curve
SVA: Sagittal Vertical Axis
SS: Sacral Slope
